# Poly-beta-amino-ester licofelone conjugates development for osteoarthritis treatment†

**DOI:** 10.1039/d3ra04967a

**Published:** 2024-01-02

**Authors:** Raed Alghamdi, Fabrizio Pertusati, Polina Prokopovich

**Affiliations:** a School of Pharmacy and Pharmaceutical Sciences, Cardiff University Redwood Building, King Edward VII Avenue Cardiff Wales CF10 3NB UK prokopovichp@cf.ac.uk

## Abstract

Disease-modifying osteoarthritis drugs (DMOADs) are a new therapeutic class for osteoarthritis (OA) prevention or inhibition of the disease development. Unfortunately, none of the DMOADs have been clinically approved due to their poor therapeutic performances in clinical trials. The joint environment has played a role in this process by limiting the amount of drug effectively delivered as well as the time that the drug stays within the joint space. The current study aimed to improve the delivery of the DMOADs into cartilage tissue by increasing uptake and retention time of the DMOADs within the tissue. Licofelone was used a model DMOAD due to its significant therapeutic effect against OA progression as shown in the recent phase III clinical trial. For this purpose licofelone was covalently conjugated to the two different A16 and A87 poly-beta-amino-ester (PBAEs) polymers taking advantage of their hydrolysable, cytocompatible, and cationic nature. We have shown cartilage uptake of the licofelone–PBAE conjugates increased 18 times and retention in tissues was prolonged by 37 times compared to the equivalent dose of the free licofelone. Additionally, these licofelone conjugates showed no detrimental effect on the chondrocyte viability. In conclusion, the cationic A87 and A16 PBAE polymers increased the amount of licofelone within the cartilage, which could potentially enhance the therapeutic effect and pharmacokinetic performance of this drug and other DMOADs clinically.

## Abstract 2

Disease-modifying osteoarthritis drugs (DMOADs) are a new therapeutic class for osteoarthritis (OA) prevention or inhibition of the disease development.

## Graphical abstract

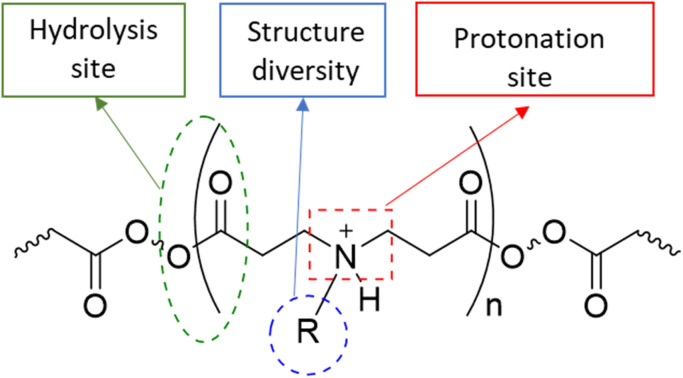

## 1 Introduction

Osteoarthritis (OA) is a chronic inflammatory disease characterised by joint stiffness and chronic pain without medication that can prevent the disease's progression.^1–6^ The current treatments are mainly symptom relief medications, such as acetaminophen, non-steroidal anti-inflammatory drugs (NSAIDs), and steroids, because OA was considered a wear-and-tear disease.^7,8^ Eventually, OA patients will seek joint replacement surgery since these medications do not prevent the disease's development. According to the national joint registry, 97% and 91% of knee and hip replacement surgeries, respectively, have been performed on patients with OA annually. Few of these patients (21 and 17%) continue to experience pain following surgery.^9,10^ The initiation cause of OA is not well known, while the progression of the disease, which involves altering the level of inflammatory cytokines, proteases, and cell activity within the synovial joint, has been investigated.^11–19^ Disease-modifying osteoarthritis drugs (DMOADs) are compounds that have been developed or repurposed to target the progression of osteoarthritis.^1–6,20,21^ Although DMOADs significantly inhibited the disease progression during pre-clinical studies, none of them have been clinically approved for OA treatment because of their insignificant therapeutic effect.^1–6,12,22–25^ The biological nature of the synovial joint has decreased the amount and duration of DMOADs within the joint, negatively affecting their clinical performance.^22–24,26^ The physiological turnover of the synovial fluid is the first obstacle, which eliminates the drug from the synovial joint and transfers it to the systemic circulation, reducing the quantity of DMOADs reaching the therapeutic target and increasing the risk of systemic adverse effects.^22,23,27,28^ The structure of the cartilage is the second obstacle, particularly for DMOAD, which has to penetrate the cartilage network to reach its therapeutic target. The passive diffusion through the cartilage is the only transport mechanism since the cartilage is avascular, alymphatic, and aneural.^29^ The cartilage is a hydrophilic condensed three-dimensional network with pore size ranging from 60 to 200 nm, thereby acting as a biological barrier that limits drugs to cross through it.^22,23,25,29–31^ Additionally, the cartilage consist of a hydrophilic negatively charge component (glycosaminoglycan), which repel negative charge molecules and attract positive charge molecules.^32^ The repulsion of the lipophilic DMOADs reduced the drug penetration and quantity inside the cartilage.^23,27,28^ Therefore, the development of DMOADs delivery systems is essential for OA therapeutics to overcome the biological challenges and to increase DMOADs' quantity and retention time within the synovial joint, enhancing their therapeutic effect, and reducing their adverse effects.^12,22–24,26,33,34^

**Fig. 1:**
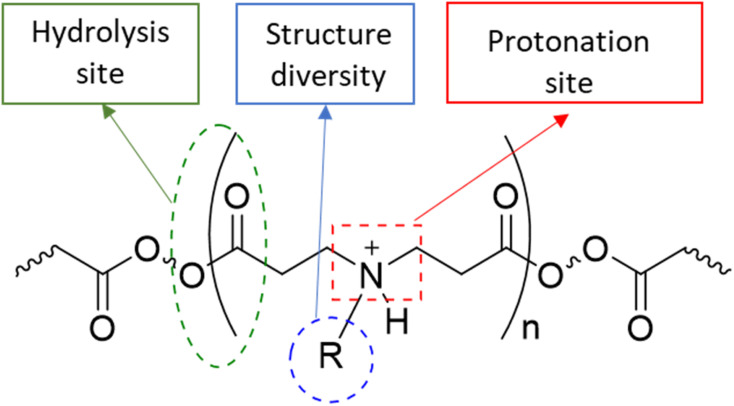
The general structure of PBAE polymers.

In this study poly-beta-amino-ester (PBAE)–licofelone conjugates were developed and characterised in terms of their uptake, retention by cartilage tissue and cytocompatibility towards chondrocytes in ex-vivo OA model. We hypothesised that the drug loaded onto the positively charged PBAE polymer will increase the quantity of the drug reaching the therapeutic target and minimise the concentration of the delivery system required.^35,36^ Cationic poly-beta-amino-ester (PBAE) polymers' structure diversity, biocompatibility, and biodegradability may provide a solution for improving DMOAD's pharmacokinetic and therapeutic effect (Fig. 1).^37–43^ The positively charge conjugates can be attracted to the negatively charged glycosaminoglycan (GAG) within the cartilage.^25,44–46^. Additionally, the PBAE structure, dimension, positive charge, charge distribution, and conjugation sites can be controlled based on the monomers,^37–41^ which is another advantage over cationic polymers that have been tested to enhance the retention time of OA therapeutics.^44,46–50^ Based on these findings, PBAE polymers were preferred over other delivery systems to investigate their influence on DMOAD residence time within the cartilage.

**Fig. 2:**
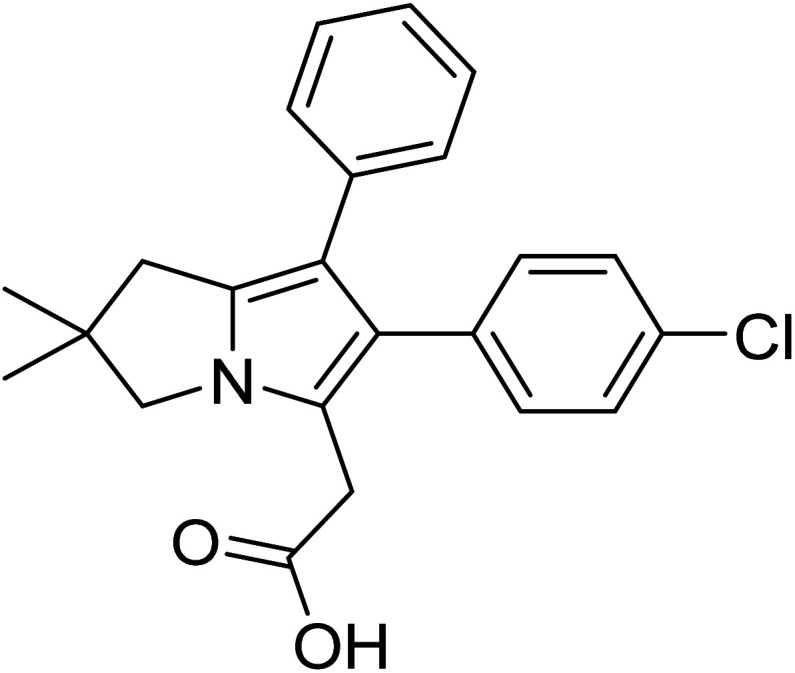
Licofelone structure.

Licofelone (Fig. 2) was chosen as a DMOAD-model to be covalently conjugated to PBAE due to its activity against OA progression by inhibiting leukotriene-B4 (LTB_4_), prostaglandin E2, and IL-1β synthesis as well as reducing iNOS level, chondrocyte apoptosis, proteases activity and expression, and osteophytes width and osteoclast count.^51–57^ Clinically, its administration (200 mg twice a day) significantly reduced cartilage loss, suppressed OA symptoms and protected against OA development, with a safe and tolerable profile.^52,58^ Additionally, this drug is currently in phase III clinical trial.^51–53,57,58^

**Fig. 3:**
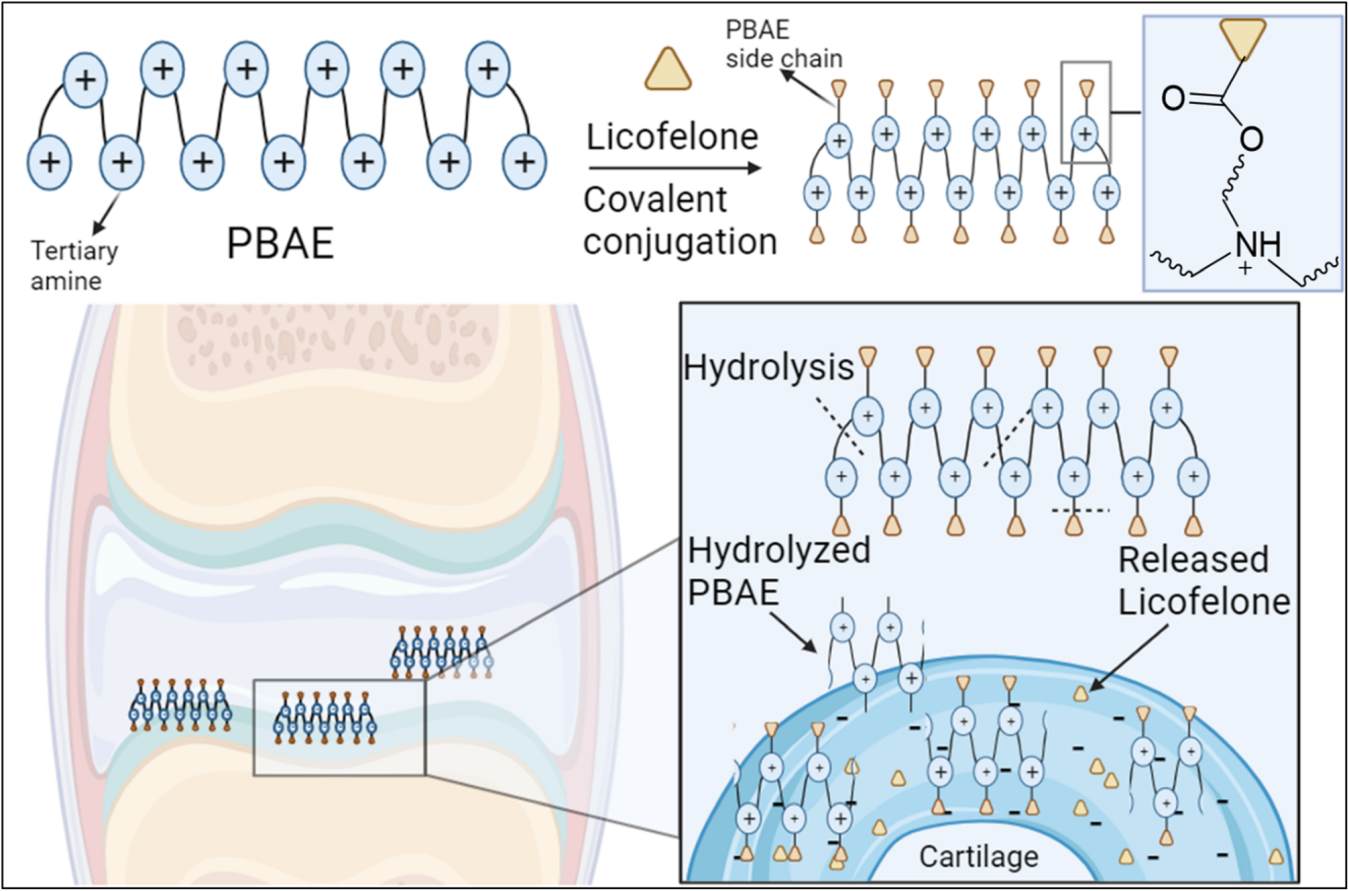
Proposed mechanism for the electrostatic interaction of PBAE–licofelone conjugate with the cartilage components. The licofelone will be conjugated to the PBAE side chain *via* hydrolysable ester bond, while the tertiary amine within the PBAE backbone will be protonated at the physiological pH 7.4. The figure created with https://BioRender.com.

The covalent conjugation of licofelone with the cationic PBAE polymers could enhance licofelone quantity and retention time within the cartilage, which will improve its therapeutic effect. The positively charged licofelone conjugates were electrostatically attracted to the negatively charged GAG, assisting the drug to penetrate the cartilage network and remain within the cartilage for an extended time and in a higher concentration (Fig. 3). Additionally, studies have reported a low acidic pH in case of knee arthritits.^39,59,60^ Therefore, the conjugates will be studied under both pH conditions, physiological pH 7.4 and inflammatory pH 5, to assist investigating the conjugates behaviour.

## 2 Experimental

### 2.1 Materials

The amine and acrylate compounds for the synthesis of PBAEs (3-amino-1-propanol (16); 1,4-butanediol diacrylate (A)), dicyclohexylcarbodiimide (DCC), *N*-hydroxysuccinimide (NHS), deuterated dimethyl sulfoxide (DMSO-d6), sodium acetate, Na_2_HPO_4_ and NaH_2_PO_4_, polyethylene glycol (PEG) standards were purchased from Sigma, UK. *N*-(3-Aminopropyl)diethanolamine (87) was purchased from Fluorochem Limited. Licofelone was obtained from Santa Cruz Biotechnology. Trypsin powder was purchased from Gibco. The cell proliferation assay kit II (XTT) by Roche was purchased from Sigma Aldrich. Solvents for the polymer synthesis and HPLC mobile phase (dichloro-methane, diethyl-ether, acetonitrile, and acetic acid glacial), and PBS were purchased from Fisher, UK. All chemicals were used as received and stored as recommended by the manufacturer.

### 2.2 PBAE synthesis

Each polymer/compound structure was confirmed by nuclear magnetic resonance (NMR) (Burker, 500 MHz, with DMSO-d6).

#### 2.2.1 Amino terminated A16 and A87 PBAE polymer preparation

The preparation protocol for PBAE synthesis was adapted from various studies.^39,41,61–63^ In a cupped glass test tube, the amino-terminated A16 and A87 were synthesised by mixing the acrylate monomer A with the amino monomer 16 or 87 in a 1.1 : 1 ratio in 5 mL of anhydrous DCM with a magnetic stirrer for 48 hours at 50 °C (oil bath). After 48 hours, heat was removed and the amino-terminated polymers were precipitated by adding 50 mL of diethyl ether or hexane. After removing the supernatant, the product was washed with 30 mL of diethyl ether or hexane to remove any remaining monomers, and then the solvent was evaporated using the rotary evaporator. The reaction schemes were reported in the ESI (Fig. S.1 and S.2†).

The ^1^H-NMR of amino terminated A16 (877 mg in diethyl ether and 1111 mg in hexane) (500 MHz, DMSO-d_6_) 4.0 ppm (8H, br, –COO–C**H**_**2**_–), 3.47–3.35 ppm (6H, m, –N–CH_2_CH_2_–C**H**_**2**_–OH), 2.6–2.7 ppm (8H, t, *J* = 6.84, 6.84, 13.68 Hz, –N–C**H**_**2**_–CH_2_–COO–), 2.42–2.35 ppm (14H, m, –OOC–C**H**_**2**_– and –N–C**H**_**2**_–), 1.6 ppm (8H, br, –OCH_2_–C**H**_**2**_C**H**_**2**_–CH_2_O–), and 1.51–1.45 ppm (6H, m, –N–CH_2_C**H**_**2**_–CH_2_–OH).

The ^1^H-NMR of amino terminated A87 (1075.4 mg in diethyl ether and 1908.1 mg in hexane) 4.0 ppm (8H, br, –COO–C**H**_**2**_–), 3.3 ppm (6H, br, –N–CH_2_–C**H**_**2**_–OH), 2.65 ppm (8H, br, –N–C**H**_**2**_–CH_2_–COO–), 2.45–2.33 ppm (14H, d, –OOC–C**H**_**2**_– and –N–C**H**_**2**_–), 1.6 ppm (8H, br, –OCH_2_–C**H**_**2**_C**H**_**2**_–CH_2_O–), 1.45 ppm (6H, br, –N–CH_2_C**H**_**2**_–CH_2_–N–).

#### 2.2.2 Acrylate terminated A16 and A87 PBAE polymer preparation

Both polymers were synthesised following the above procedures except that the acrylate monomer was mixed with the amino monomer in a 1.1 : 1 ratio. The reaction scheme with the ^1^H-NMR chemical shift analysis were reported in ESI (Fig. S.1 and S.2†). The ^1^H-NMR spectra of acrylate-terminated A16 and A87 polymers were used to determine the average molecular weight (*M*_w_) of these polymers based on the method developed by Paulsen and Frasco.^64^ The samples were prepared at a concentration of 10 mg mL^−1^ of DMSO-d6.

### 2.3 A16 and A87–licofelone conjugates synthesis

In a 50 mL round bottom flask, 110 mg of amino-terminated polymer, 53 mg of licofelone (3 equivalents), 64 mg of DCC (6.6 equivalents), and 64 mg of NHS (6.6 equivalents) were mixed in 25 mL of anhydrous DCM (Fig. S.3†). The conjugation reaction was performed at 0 °C for the first 30 minutes, then at room temperature for the next 48 hours under argon and stirring. After 48 hours, the reaction solution was filtered using filter papers (185 mm) to remove the white precipitate of dicyclohexylurea (DCU). Then, 200 mL of hexane was poured to precipitate A16–licofelone and A87–licofelone conjugates. The precipitates of A16–licofelone and A87–licofelone conjugates were washed with hexane three times; after each wash, the supernatant was discarded. A16–licofelone and A87–licofelone conjugates were dried using a rotary evaporator for 45 minutes, and the products were analysed by ^1^H-NMR (500 Hz, DMSO-d_6_).

The ^1^H-NMR of A16–licofelone conjugate (102.4 mg); aromatic rings protons 6.95–7.36 ppm (10H, m), 4.0 ppm (14H, s, –COO–C**H**_**2**_–), 3.7 ppm (1H, s, –N–C**H**_**2**_–C), 3.47 ppm (1H, s, –C–C**H**_**2**_–C=C), 3.3 ppm (6H, m, –N–CH_2_CH_2_–C**H**_**2**_–OH), 2.9 ppm (2H, br, –C**H**_**2**_–COO–CH_2_), 2.7–2.8 ppm (8H, br, –N–C**H**_**2**_–CH_2_–COO–), 2.35–2.48 ppm (14H, br, –OOC–C**H**_**2**_– and N–C**H**_**2**_–), 1.6 ppm (8H, br, –OCH_2_–C**H**_**2**_C**H**_**2**_–CH_2_O–), 1.4 ppm (6H, br, –N–CH_2_C**H**_**2**_–CH_2_–OH), and 1.23 ppm (6H, s, C**H**_**3**_–C–C**H**_**3**_).

The ^1^H-NMR of A87–licofelone conjugate (111.8 mg); aromatic rings protons 6.95–7.36 ppm (10H, m), 4.0 ppm (20H, br, –COO–C**H**_**2**_–), 3.7 ppm (1H, s, –N–C**H**_**2**_–C), 3.47 ppm (1H, s, –C–C**H**_**2**_–C=C), 2.7 ppm (2H, br, –C**H**_**2**_–COO–CH_2_), 2.6–2.7 ppm (8H, br, –N–C**H**_**2**_–CH_2_–COO–), 2.3–2.4 ppm (14H, br, –OOC–C**H**_**2**_– and –N–C**H**_**2**_–), 1.6 ppm (8H, br, –OCH_2_–C**H**_**2**_C**H**_**2**_–CH_2_O–), 1.4–1.5 ppm (2H, m, –N–CH_2_C**H**_**2**_–CH_2_–N–), and 1.23 ppm (6H, s, C**H**_**3**_–C–C**H**_**3**_).

### 2.4 PBAE polymer and conjugates characterisation

Each data value was an average of three measurements on three independent batches.

#### 2.4.1 The average molecular weight determination

The average molecular weight of A16 and A87 polymers were determined using Gel Permeation Chromatography (GPC, Shimadzu, RID-20A) based on PEG standards. 1 mg mL^−1^ of PEG (200–36 000 Da) was dissolved in pH 5 to build a calibration curve (Fig. S.4†). The GPC system was equipped with Superdex™ 75, 10/300 GL column using 100% of AcOH/NaOAc pH 5 as a mobile phase at flow rate of 1 mL min^−1^ and refractive index detector. The polymers were dissolved at PBS buffer pH 7.4 and acetate buffer pH 5 at 2 mg mL^−1^.

#### 2.4.2 Zeta potential and hydrodynamic size determination

The electrophoretic mobilities of the polymers, licofelone, and licofelone conjugates was measured using the Malvern Zetasizer Nano ZS (Malvern Instruments Ltd, Malvern, UK) at the physiological pH 7.4 and the inflammatory pH 5. The PBAE polymers and conjugates were prepared at 2 mg mL^−1^, while licofelone was prepared at 1 mg mL^−1^ 1 mL of the solution was transferred into folded capillary zeta cell. Zeta potentials were calculated from electrophoretic mobility using the Smoluchowski model.

### 2.5 Licofelone quantification method

Reverse phase-HPLC (RP-HPLC, Shimadzu, LC-2030C Plus) (Fig. S.5†) was used to quantify the amount of the licofelone in the buffer and the cartilage digested buffer. The system was equipped with a C18 column (Kinetex 5 μm C18 100 Å, LC Column 250 × 4.6 mm) and UV-detector, which was at 248 nm. The mobile phase consisted of 50% PBS pH 7.4 and 50% ACN at a 1 mL min^−1^ flow rate. The licofelone conjugate samples were prepared at a concentration of 3 mg mL^−1^ in a mixture of pH 7.4 PBS and DMSO (75 : 25, v : v). 1 mL of the solution was transferred into HPLC vail for quantification based on the licofelone standard calibration curve using the RP-HPLC method. 10 μL of pH 7.4 PBS and DMSO (75 : 25, v : v) was added to replace the volume that was injected in the HPLC. The quantity of the released licofelone was determined for up to 120 hours.

### 2.6 The harvest of the bovine articular cartilage

The cartilage were harvested from the metacarpophalangeal joint (MCP joint) of an immature (6–8 days old) bovine steer feet, which were collected from a local abattoir (Drudy and Sons Abattoir, Tockenham, Swindon, UK). A 5 mm biopsy puncher was used to obtain cylindrical cartilage discs with a 5 mm diameter under sterile conditions.^63^ GAG-depleted cartilage samples (model of early OA stage) were prepared by digesting the cartilage in 500 μL of a PBS solution of trypsin (1 mg mL^−1^) for 24 h at 37 °C followed by washing three times with fresh PBS. The preparation of buffers, cartilage digestion solution, and the cartilage complete media were described in the ESI.†

### 2.7 The *ex vivo* bovine cartilage model of early OA stage

Cartilage samples were extracted as described before and weighted to normalise the results. The disks were equilibrated in serum-free medium (low-glucose DMEM, l-glutamine, 25 mM HEPES, 110 mg L^−1^ sodium pyruvate), supplemented with 1% insulin–transferrin–selenium (corresponding to insulin 10 μg mL^−1^, transferrin 5.5 μg mL^−1^ and selenium 5 ng mL^−1^), 0.1 mM nonessential amino acids (Gibco, UK), 4 M proline (Sigma Aldrich, UK), 20 mg mL^−1^ ascorbic acid (Fisher Scientific, UK), 100 units per mL penicillin G, 100 μg mL^−1^ streptomycin and 250 μg mL^−1^ amphotericin B (Sigma Aldrich, UK) for two days prior to treatment at 37 °C in 5% CO_2_. Cartilage samples (*n* = 6) were incubated at 37 °C in a humidified atmosphere containing 5% CO_2_; the medium was changed every 2 days.

To simulate the early OA stage, cartilage was treated with 1 mg mL^−1^ trypsin in PBS buffer for 24 hours at 37 °C to induce GAG loss, and then the cartilage content (GAG and collagen) was measured.^39,65,66^ The GAG content was measured using dimethyl-methylene blue (DMMB) reagent assay^67^ while the collagen content was determined using the hydroxyproline assay.^68^

### 2.8 Uptake and retention study

For the uptake study, the 5 mm cartilage discs were sliced in half and weighted. The half-sliced cartilage was placed in a transport chamber that was designed to perform a one way diffusion study into the cartilage as described in ref. 36. A 50 μL of licofelone, A16–licofelone, or A87–licofelone was added in the chamber facing the cartilage superficial side, while the deeper side (the closest to the bone) was filled with 50 μL of PBS buffer pH 7.4. The transport chamber was placed in a closed Petri dish containing water and incubated for 1–30 minutes at 37 °C. At required intervals (0.5, 1, 2, 3, 5, 7, 10, 15 and 20 min), cartilage samples were removed, washed in plentiful amounts of water and placed in an Eppendorf tube containing 1 mL of digestion buffer for 48–96 hours in a 50 °C oven. For the retention study, the cartilage was treated with 50 μL of licofelone or licofelone conjugates formulation, following the method described above, for 20 minutes at 37 °C. Then, the cartilage were dipped and placed in Eppendorf vails containing 500 μL PBS buffer pH 7.4 for 1–60 minutes at 37 °C, while the A87–licofelone study was extended to 120 minutes because the licofelone was detectable after 60 minutes. After incubation, the cartilage was transferred to an Eppendorf vail containing 1 mL of digestion solution for 48–96 hours at 50 °C. Licofelone was prepared at a concentration of 1 mg mL^−1^ of PBS buffer pH 7.4/DMSO in a 1 : 1 ratio, while the licofelone conjugates were prepared at 4 mg mL^−1^. The DMSO has no effect on the uptake and retention time studies (Fig. S.6 and S7†). The results of the uptake and retention time studies are represented as a percentage of the drug quantity, which was calculated based on eqn (1) to allow an accurate comparison between the studies. Additionally, the area under the curve was calculated to determine the total percent of the uptake and the retained drug inside the cartilage over time.1
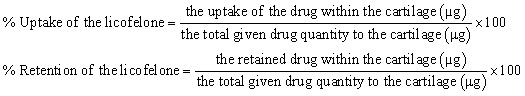


A phosphate buffer (0.2 M pH = 6.8) containing 300 mg L^−1^ of papain, 1 mM EDTA and 2 mM dithiothreitol (DTT) was used to digest the cartilage. The cartilage tissues were placed in 1 mL of the digestion buffer and incubated at 55 °C for 24 h.

All experiments were performed on both the normal cartilage (healthy condition) and the GAG depleted cartilage (mimicking the early stage of OA). Determinations were performed on triplicate samples originating from 3 different bovine animals using three independent batches of emulsions.

### 2.9 XTT tissue proliferation assay

The XTT assay reagent (*in vitro* toxicology assay kit (Sigma-Aldrich, UK)) was used to determine cartilage tissue viability. The XTT cell viability experiment was performed in accordance with the manufacturer's instructions with minor modifications. Fresh cartilage discs were harvested as previously mentioned, and then placed in a 48-well plate containing 500 μL cartilage complete medium for 72 hours in a 37 °C and 5% CO_2_ incubator. Then, the medium was discarded, and the cartilage discs were treated with 500 μL of licofelone, A87 polymer, A87–licofelone conjugate, or complete medium (control) for 24 or 48 hours at 37 °C and 5% CO_2_. After the treatment, the supernatant was discarded, and the cartilage was washed with 500 μL of fresh medium, sliced into 3 pieces and incubated with 500 μL XTT solution for 4 h at 37 °C in 5% (v/v) CO_2_ in air. Then, the XTT solution was removed and retained to be used later. After that, 250 μL of dimethyl sulfoxide (DMSO) were added and incubated for 1 h to extract the tetrazolium product from the tissue. XTT and DMSO solutions were then mixed before reading the absorbance of triplicate samples at 450 nm and 690 nm respectively in a 96 well plate using a spectrophotometer (Tecan, Infinite 200 PRO). Absorbances at 690 nm were subtracted from the absorbances measured at 450 nm and the XTT content was calculated per gram of cartilage tissue.

### 2.10 Statistical analysis

One-way or two-way ANOVA was performed between groups; for multiple comparisons followed by Tukey or Dunnett post hoc test.^69^*T* test was performed to compare between two groups. The data is reported as the mean ± standard deviation (SD) of three or more independent experiments. The area under the curve (AUC) was calculated by the trapezoid rule.^70^ All the experiments were conducted in triplicate, unless stated otherwise. The statistical analysis was performed using IBM SPSS Statistics (Version 25).

## 3 Results and discussion

### 3.1 Polymers and conjugates characterization

**Fig. 4:**
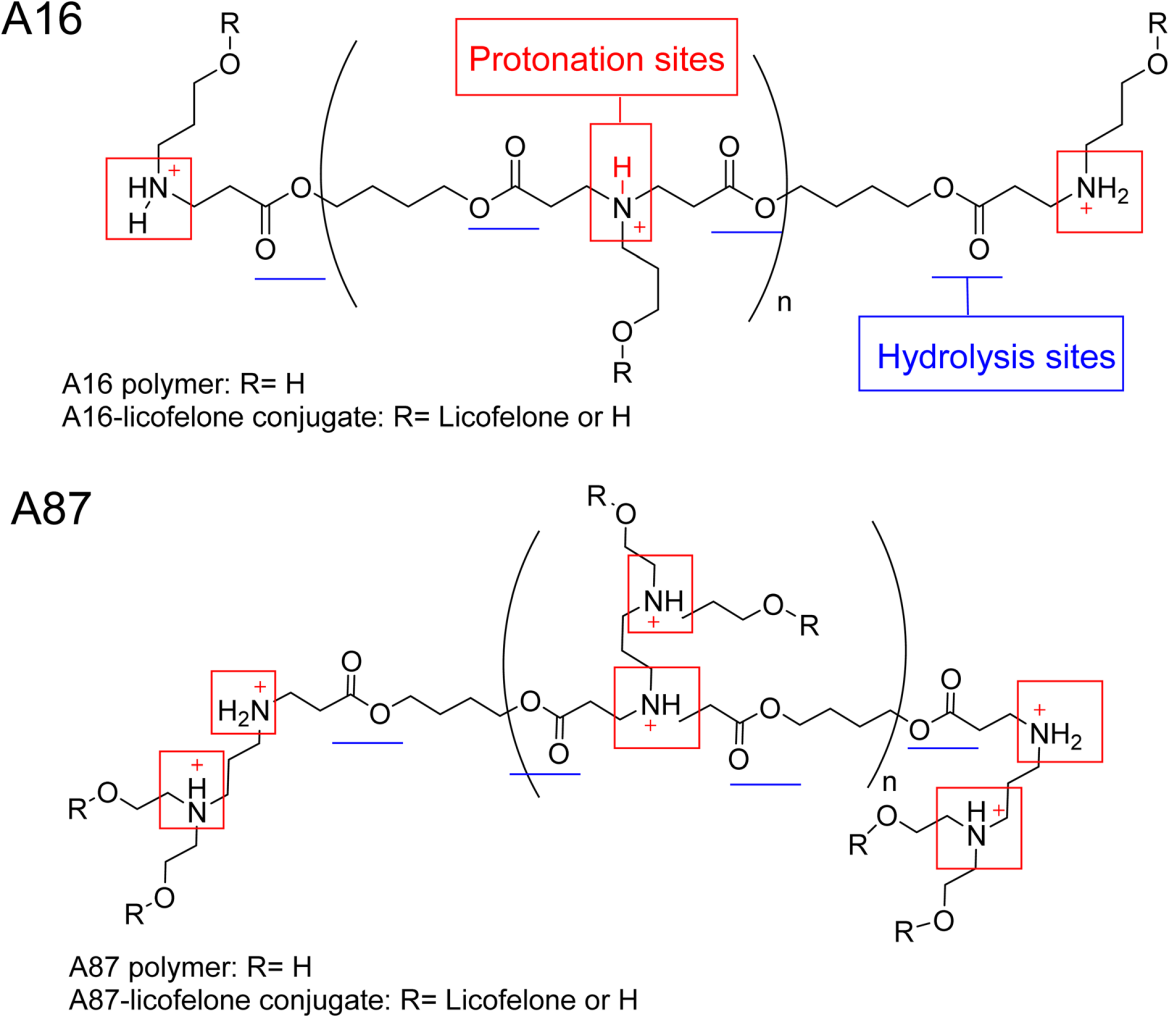
The chemical structure of A16, A87, and their respective licofelone conjugate.

The ^1^H-NMR spectra of A16 and A87 polymers were identical to those reported in the literature.^37,43,61,71,72^ The signals at 2.63–2.66 ppm and 2.42–2.35 ppm represented the new bond formation between monomers, and the typical acrylate protons located at 6.3, 6.18, and 5.9 ppm were the key signals that differentiated between the formation of acrylate and amino-terminated polymers (Fig. S.8†). The conjugation was confirmed by the appearance of licofelone aromatic proton signals at 6.95–7.4 ppm as well as the disappearance of the carboxylic acid signal at 12.5 ppm (Fig. S.9†). The precipitation of both polymers was higher in hexane, which may be related to the polymers' polarity (Fig. 4). Therefore, the conjugates of licofelone were precipitated in hexane only.

The average *M*_w_ of both polymers was similar to those of other PBAE polymers reported in the literature.^40,61^ The average *M*_w_ of PBAE polymers were ranging between 2000 and 120 000 Da. A16 and A87 were designed to have a relatively small *M*_w_ to increase the penetration through the cartilage mesh.^25,40,41^ Furthermore, A16 and A87 showed a positive net charge in both pH due to the tertiary amines protonation in the backbone of the polymer (Fig. 4). At pH 5, the A87 showed the highest positive charge (13.8 ± 1.7 mV) because it contained two tertiary amines in each repeating units, which were highly protonated as the pH dropped (Table 1). The polymer positive charge variation further assisted in investigating the effect of the positive charge degree on the conjugated licofelone uptake and retention study. The surface net charge of the A16 and A87 increased after the conjugation reaction from 7.8 ± 0.5 and 6.9 ± 0.8 mV to 15.2 ± 2.72 and 12.25 ± 0.61 mV, respectively, thereby the conversion of the hydroxyl groups to ester, which was a neutral functional group (Table 1). Additionally, the change of the polymers' surface charges was another conformation of the conjugate formation. As previously mentioned, the conjugation of DMOADs with the cationic PBAE would hide the undesirable physiochemical properties of the drug, which was observed with licofelone. The licofelone is a lipophilic molecule with a negative surface charge (−9.69 ± 2.69 mV) that is insoluble in aqueous solution, while the licofelone conjugates are cationic and soluble in pH 7.4 and pH 5.

**Table 1:** Characterization of A16 and A87 polymers (mean ± SD; *n* = 3)

PBAE polymer	Zeta potential (mV)		GPC average *M*_w_ (Da)		NMR repeating unites (*n*)	NMR average *M*_w_ (Da)
	pH 7.4	pH 5	pH 7.4	pH 5		
A16	7.8 ± 0.5	7.1 ± 0.5	1138.3 ± 55.9	1315 ± 103.9	6.6 ± 0.3	2010.7 ± 76.4
A87	6.9 ± 0.8	13.8 ± 1.7	1756.3 ± 260.3	1739.3 ± 95.4	5.9 ± 0.4	2324.8 ± 154.8

The PBAE were linked by an ester bond, which was responsible for the rapid hydrolysis that occured within hours (Fig. 4). Additionally, the polymers previously showed faster hydrolysis at pH 7.4 than pH 5.^40–42,73^ A quick hydrolysis during the first 8 hours was observed in the A16 and A87 hydrolysis study, which was similar to the values reported in the literature (Fig. S.10†).^40–42,73^ A16 polymer average *M*_w_ at pH 5 was numerically higher when compared to pH 7.4 and the *M*_w_ was significantly reduced after 2 hours at pH 7.4 and after 3 hours at pH 5 (Fig. S.10A†). Furthermore, the average *M*_w_ of A87 at pH 5 was numerically higher than pH 7.4 with respect to time point and showed a statistically higher average *M*_w_ at 6 and 7 hours (Fig. S.10B†). The hydrolysis study of A16 and A87 indicated that the polymers were degraded faster at pH 7.4 than at pH 5. The advantage of developing a degradable delivery system is to ensure that the DMOADs would be fully released and not trapped within the delivery system. Additionally, a fast released DMOADs would potentially has an immediate therapeutic activity.^74^

### 3.2 Quantification of the released licofelone

**Fig. 5:**
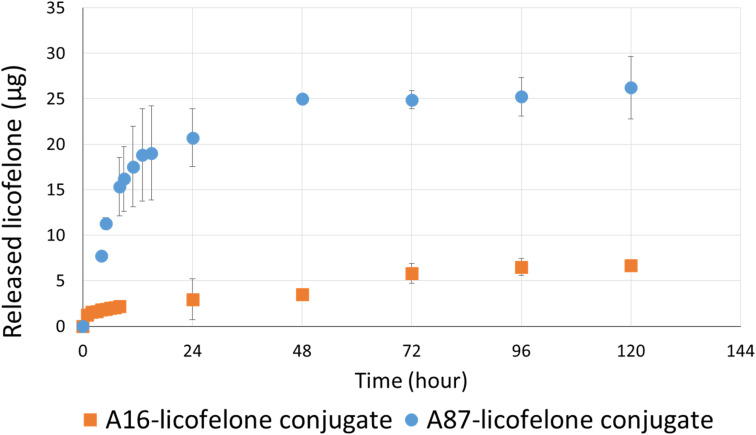
The released licofelone quantity over time (0–120 hours) from A16–licofelone conjugate and A87–licofelone conjugate; bars represent (mean ± SD of *n* = 3 for A16–licofelone, *n* = 4 for A87–licofelone).

The RP-HPLC method determined the quantity of licofelone and licofelone released by the polymer, cartilage uptake, and cartilage retention studies at a minimum of 0.1 μg. One of the objectives of this study aimed to investigate the influence of conjugated licofelone quantity on the drug uptake and retention within the cartilage. A87 contains two conjugation sites in each repeating unite, while A16 contains a single conjugation site in each repeating unite, which mean A87 polymer should contain a higher licofelone quantity. The release study showed that the released licofelone from 3 mg mL^−1^ of A16–licofelone conjugate was 6.7 ± 0.2 μg and from 3 mg mL^−1^ of A87–licofelone was 26.2 ± 3.4 μg (Fig. 5). The release study also showed that a full release of conjugated licofelone was observed at 48 and 96 hours from the A87 and A16 polymers, respectively. The fact that A16–licofelone had a slower release profile than A87–licofelone could be related to the significantly higher loading of A87, which is 4 times higher than A16 polymer. Additionally, another study reported that dexamethasone–avidin conjugate *via* an ester bond, was able to release 50% of the drug 14.4 ± 1 hours at pH 7.4, supporting the current study findings.^44^ Furthermore, the HPLC chromatogram showed that the released licofelone had a similar chemical structure to the standard licofelone since both molecules had a similar retention time (Fig. S.11†).

### 3.3 The cartilage uptake and retention study

**Fig. 6:**
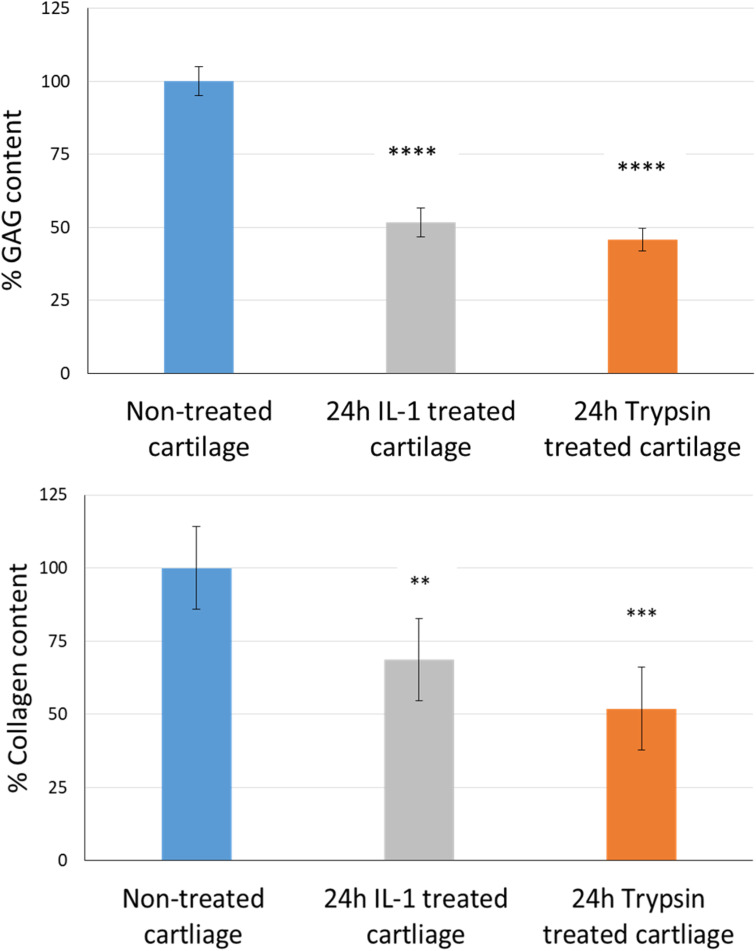
The percent of GAG and collagen contents; GAG bars represent (mean ± SD of *n* = 3) and collagen content bars represent (mean ± SD of *n* = 5). Significant ** (*P* < 0.01), *** (*P* < 0.001), and **** (*P* < 0.0001) compared to the control.

The uptake and retention time of free licofelone and conjugated licofelone was determined in the healthy and OA cartilage *ex vivo* models. The OA model was developed to investigate the effect of cartilage loss on the efficiency of A16–licofelone and A87–licofelone in delivering the drug. For this reason that the delivery system depended on the electrostatic interaction between the conjugate and the GAG content to penetrate and to retain within the cartilage. The trypsin treated cartilage (OA cartilage) showed approximately 50% depletion of GAG and collagen contents, which mimicked an early stage of osteoarthritic cartilage (Fig. 6).^39^

**Fig. 7:**
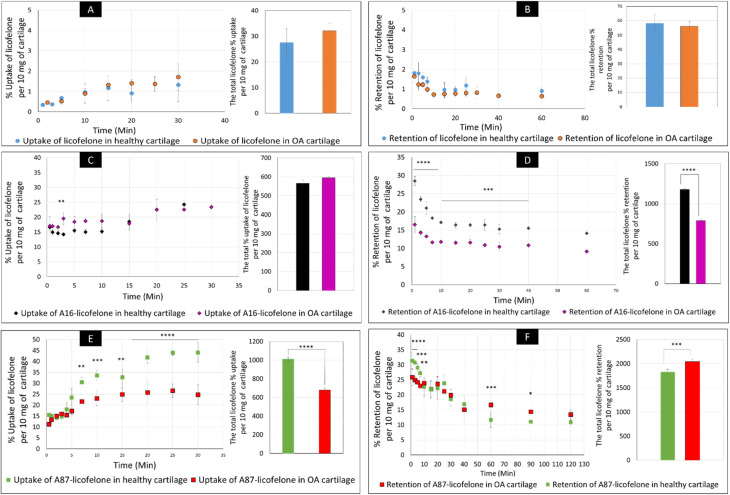
The uptake and retention time of free licofelone and conjugated in both cartilage models. The graphs (A) and (B) showed the free licofelone uptake and retention time in both cartilage, respectively. The (C) and (D) showed the uptake and retention time of conjugated licofelone to A16 in both cartilage models, respectively. The graphs (E) and (F) showed the uptake and retention time of conjugated licofelone to A87 in both cartilage models, respectively. The bars represented the total uptake and retention of licofelone in its respective study. The data represented as (mean ± SD of *n* = 3). Significant * (*P* < 0.05), ** (*P* < 0.01), *** (*P* < 0.001), **** (*P* < 0.0001).

The percent uptake of free licofelone and conjugated licofelone has increased in a time-dependent manner in both cartilage models (Fig. 7). The percent uptake of licofelone alone was significantly low, reaching a maximum uptake of 1.3 ± 0.8 and 1.7 ± 0.6% in healthy and OA cartilage with no statistical different between both models. The low uptake percent was expected since the negatively charged cartilage would repeal the negatively charged and lipophilic licofelone.^32^ Numerically, the percent uptake of licofelone was higher in OA cartilage, which was related to the 50% cartilage content depletion. This result supported the hypothesis that the cartilage was acting as a biological barrier, preventing therapeutics from infiltrating through.^23,27,28^ Similar results were observed in the retention study of licofelone in both cartilage models, with an extremely low total percent of licofelone retained over 60 minutes in healthy cartilage (58.3 ± 6.2%) and in OA cartilage (56.0 ± 3.2%). The comparison of the licofelone conjugates between the two cartilage models showed a high percent of the conjugated drug uptake and retention in healthy cartilage when compared to the OA cartilage model (Fig. 7). The uptake of conjugated licofelone to A16 showed no difference between the two cartilage models, but the retention was statistically higher in the healthy cartilage model, in which the total retained conjugated licofelone was 1179 ± 11.8% in healthy cartilage, while it was 787.2 ± 8.9% in the OA cartilage model. Moreover, the retention percent of conjugated licofelone was significantly greater at all minutes in the OA model (Fig. 7D). In addition to the A16–licofelone conjugate, A87–licofelone showed a statistically significant enhancement of licofelone uptake and retention in the healthy cartilage model compared to the OA model. A 7.9 to 19.3% increase of licofelone uptake was observed in the healthy cartilage from 7 to 30 minutes compared with OA cartilage models (Fig. 7E). Although the total retained percent of licofelone was significantly higher in the OA model (2040 ± 57%) than the healthy model (1829 ± 54.7%), the retained percent was significantly higher during the first 10 minutes in the healthy model (Fig. 7F). Then, the percent of conjugated licofelone was comparable in both cartilage models during 15 and 40 minutes. At 60 and 90 minutes, the retained percent of licofelone was higher in the OA model. Overall, the high percentage of conjugated licofelone uptake and retention in the healthy cartilage compared to the OA cartilage models confirms that the delivery system is charge-based. The positively charged A16 and A87 licofelone conjugates were electrostatically attracted to the negatively charged GAGs, which has increased the uptake and retention time of licofelone in healthy cartilage more than in the GAG depleted cartilage, which supported the current study hypothesis.

**Fig. 8:**
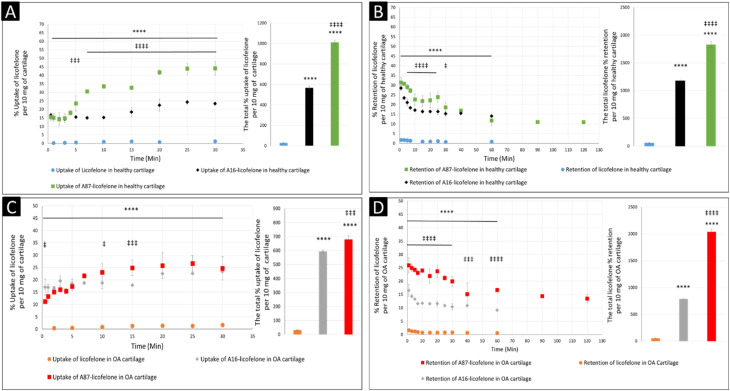
The comparison of licofelone and conjugated licofelone uptake and retention in both cartilage models. The (A) and (C) graphs showed the uptake comparison in healthy cartilage and the early-simulated OA cartilage model, respectively. The (B) and (D) graphs showed the retention comparison in the healthy cartilage and OA cartilage models, respectively. The bars represented the total uptake and retention of licofelone in its respective study. The data represented as (mean ± SD of *n* = 3). Both licofelone conjugates were significant **** (*P* < 0.0001) when compared to the free licofelone, while the A87–licofelone conjugate was significant ‡ (*P* < 0.05), ‡‡‡ (*P* < 0.001), and ‡‡‡‡ (*P* < 0.0001) compared to the A16–licofelone conjugate.

The negatively charged GAG could be used as an advantage to assist therapeutics to infiltrate and be retained within the cartilage if a positively charged drug delivery system was adapted, which was observed after conjugating licofelone to A16 and A87.^45,46^ The percentage of conjugated licofelone penetrated and retained within the condensed cartilage mesh network was significantly increased when compared with the free licofelone (Fig. 8). At 30 minutes, a maximum uptake of conjugated licofelone to A16 and A87 has reached 23.4 ± 0.85 and 44 ± 4.3% in healthy cartilage and 23.4 ± 0.3 and 26.6 ± 3.1% in OA cartilage, respectively, which is statistically higher than the unconjugated licofelone. Additionally, the total uptake of licofelone was statistically increased from 27.5 ± 5.2% in healthy cartilage and 32.1 ± 2.9% in OA cartilage to 566.2 ± 16.75 and 1012 ± 22.4% in healthy cartilage, as well as 595 ± 8.4 and 679.6 ± 28.7% in OA cartilage after conjugation to A16 and A87, respectively. Furthermore, the total licofelone retained (in the healthy model and 56.0 ± 3.2% in the OA model) was significantly enhanced after conjugation to A16 and A87 (1179 ± 11.8 and 1829 ± 54.7% in healthy cartilage, while 787.2 ± 8.9% and 2040 ± 57% in OA cartilage). The electrostatic interaction between the conjugated systems and the GAG has enhanced the drug residence time and quantity within both cartilage models. Moreover, the A16 and A87 polymers have masked the undesirable physiochemical properties of licofelone, which has minimised the cartilage-repellence activity against the drug. The results showed that the positively charged conjugates have increased the licofelone resident time within the cartilage compared to the licofelone alone, unfortunately the comparison against neutral PBAE conjugate wasn't possible because the presence of the tertiary amines in the backbone of the polymer (Fig. 1). However, a study has reported that positive avidin has infiltrated the cartilage faster and deeper than neutral avidin, which supports the current study's finding.^45^ Furthermore, the cationic polyamidoamine (PAMAM) dendrimers increased the uptake of insulin-like growth factor 1 (IGF-1) by 51 and 71% in the full healthy cartilage disc after 24 hours of incubation, while A87 increased the licofelone uptake by 44% in the half-cartilage disc after 30 minutes of incubation.^48^ The high quantity of conjugated licofelone within the cartilage could enhance the pharmacokinetics and therapeutic effect of the drug, which was previously discussed in other literature.^47,75–78^ Licofelone activity as DMOADs such as inhibiting chondrocyte apoptosis, inhibiting the expression of MMP-1, MMP-13, ADAMTS-5, and cathepsin K, reducing the osteoclast count and reducing the width of osteophytes could be related to mechanism other than COX-2 and 5-LO inhibition, which requires the presence within the cartilage.^51–57^ Therefore, increasing the residence time of licofelone within the cartilage will enhance the drug performance therapeutically.

The comparison of A16–licofelone conjugate to A87–licofelone has revealed two factors that play a role in developing an efficient delivery system for OA therapeutics. The first factor is the degree of the positive charge, which was reported previously. Although the positive charge of A16–licofelone was higher than A87–licofelone conjugate, A87–licofelone significantly increased the conjugated licofelone uptake and retention in both cartilage models compared with A16–licofelone (Fig. 8). According to the current study hypothesis, the positive charge should be proportional to the increase in drug uptake and retention. However, a study on various positively charged peptides has reported that +16 peptides and higher were strongly attracted to the cartilage component on the surface, which limited the penetration.^46,49^ This could be the reason for reduced A16 conjugate uptake and retention compared to A87 conjugate. Therefore, the positive charge should be a control to maintain the intensity of the electrostatic interaction. Furthermore, the quantity of licofelone that was loaded on A87 was 4 times higher compared with A16, which could simultaneously play a role in increasing the uptake and retention of conjugated licofelone to A87 more than A16.

### 3.4 Cartilage tissue viability

**Fig. 9:**
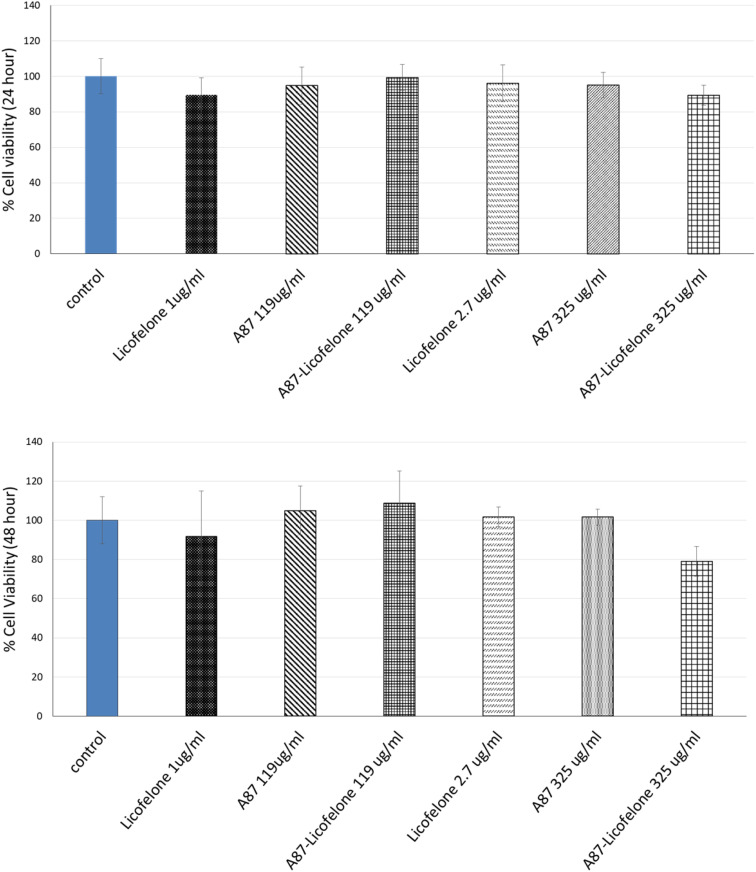
The effect of licofelone, A87 polymer, and A87–licofelone on cartilage tissue viability after 24 and 48 hours of treatment.

The cartilage tissue viability assay was performed only using the A87–licofelone conjugate because of its preferable results compared to the A16–licofelone. The A87–licofelone concentration was chosen based on the uptake and retention study. The viability assay was conducted at 1 μg of licofelone, which was the maximum uptake of the conjugated licofelone quantity within the cartilage, and at 2.7 μg, which was the highest quantity of licofelone applied to the cartilage. According to the release study, these quantities would be at 119 and 325 μg mL^−1^ of A87–licofelone. Additionally, 1 and 2.7 μg of standard licofelone and 119 and 325 μg mL^−1^ of A87 polymer alone were used as controls. Fig. 9 showed the cartilage viability of the compounds after 24 and 48 hours of treatment. At 24 and 48 hours, all concentrations showed no statistically significant difference in cell viability compared to the untreated cartilage tissue. In previous studies PBAE polymers showed no toxicity against various cell lines.^41–43^ Additionally, licofelone has been a safe drug with no side effects and has been well tolerated during *in vivo* and clinical studies.^51–58^

## 4 Conclusions

The covalent conjugation of A87 and A16 to licofelone enhanced the drug uptake and retention in both cartilage models, which would significantly enhance licofelone's performance therapeutically and pharmacokinetically. The conjugation of licofelone to A16 and A87 had no effect on the chemical structure and the toxicity of licofelone in cartilage tissue. The A87 polymer should be further investigated with other clinically relevant DMOADs such as kartogenin, rhein, cindunistat, diacerein, and AGG-523. The application of PBAE polymers, and particularly A87, should be further investigated as a delivery system for other DMOADs.

## Conflicts of interest

PP is a named inventor in the patent application related to the application of PBAE to cartilage treatment. FP and RA have no conflict to declare.

## Supplementary Material

RA-014-D3RA04967A-s001
