# Supplementary material for: Poly-beta-amino-ester licofelone conjugates development for osteoarthritis treatment
Source: RSC Adv. 2024 Jan 2;14(1):15–28. doi: 10.1039/d3ra04967a (PMC10758810; doi:10.1039/d3ra04967a)
Supplement: RA-014-D3RA04967A-s001 [file RA-014-D3RA04967A-s001.pdf]

Electronic Supplementary Material (ESI) for RSC Advances.
This journal is © The Royal Society of Chemistry 2023

Supplementary information for

Poly-beta-amino-ester licofelone conjugates development for osteoarthritis treatment.

Raed Alghamdi [a], Fabrizio Pertusati [a], Polina Prokopovich[a]*

[a] School of Pharmacy and Pharmaceutical Sciences, Cardiff University, Cardiff, Wales, United Kingdom

Experimental procedure

Materials

Cartilage media materials that were purchased from Thermo Fisher Scientific are Dulbecco's Modified Eagle Medium (DMEM, 11885-084), insulin-transferrin-selenium, and minimum non-essential amino acids (MEMNEAA) by Gibco, while penicillin/streptomycin, amphotericin B, and proline were purchased from Sigma Aldrich.

Buffers preparation

Phosphate buffered saline (PBS, pH 7.4) was prepared by dissolving a 100 g tablet of PBS in 100 ml of deionized water (dH_2O). The sodium acetate buffer (pH 5) was obtained by mixing (volume to volume) 30% of (5.8 ml acetic acid in 994.2 ml of dH_2O) with 70% of (13.6 g sodium acetate trihydrate in 1000 ml of dH2O).

Cartilage digestion solution

The digestion buffer was prepared by mixing 2 mM of DTT and 0.3 mg/ml of papain in a pH 6.8 buffer, which contains 20 mM sodium phosphate buffer and 1 mM of EDTA.(1) The 1 ml of digestion solution contains 900 µl digestion buffer and 100 µl DMSO.

Preparation of cartilage complete medium

The complete medium contains 500 ml DMEM, 5 ml insulin-transferrin-selenium, 5 ml penicillin/streptomycin, 5 ml MEMNEAA, 0.5 ml amphotericin B, 0.5 ml ascorbic acid, and 0.5 ml proline. The 250 µg of amphotericin B, 20 mg of ascorbic acid, and 4 moles of proline were separately dissolved in 1 ml of sterile PBS pH 7.4.(2)

The XTT assay sample preparation

0.135 % DMSO was used to solubilize 1 and 2.7 µg/ml licofelone, whereas the A87-licofelone and A87 polymer were soluble in complete medium. Therefore, the cytotoxicity of 0.135 % DMSO was investigated. At 24 and 48 hours, 0.135 % DMSO-treated cartilage showed no statistical difference compared to the untreated cartilage (control) (Figure S. 6), so no toxicity associated with the use of 0.135 % DMSO was observed.

Figures

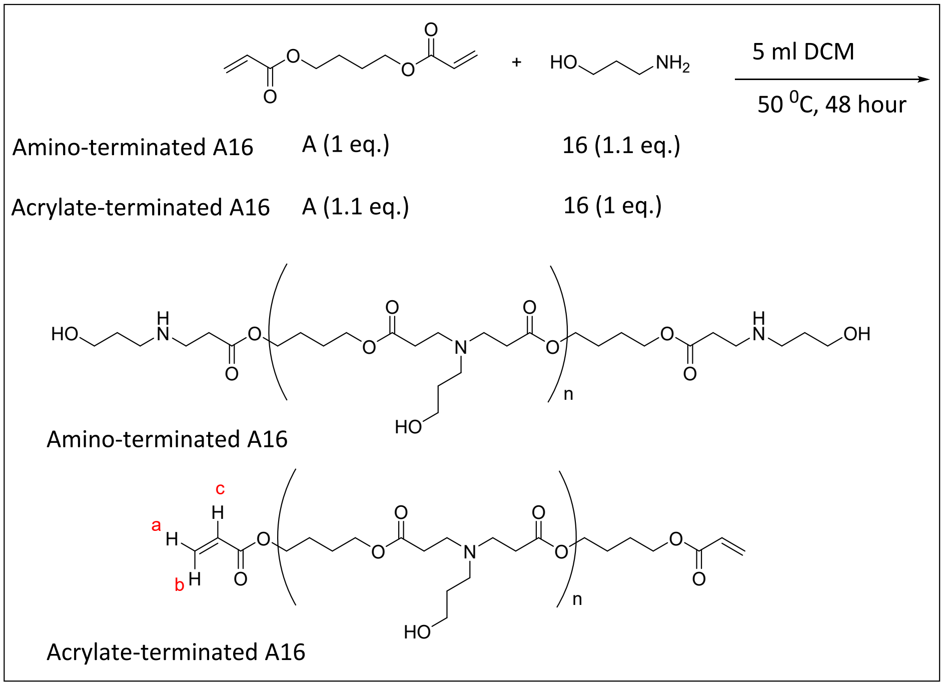

*Figure S.1: The synthesis of amino and acrylate-terminated **A16***

The ^{1}H-NMR of the acrylate-terminated **A16** 6.3 ppm (2H, d, J= 17.73 Hz, CH_a $\mathbf{H_b}$=CH_c-), 6.1 ppm (2H, dd, J= 10.36, 10.36, 17.73 Hz, CH_a H_b=$\mathbf{CH_c}$-), and 5.9 ppm (2H, d, J= 10.36 Hz, $\mathbf{CH_a}$ H_b=CH_c), 4.0 ppm (8H, br, -COO-$C\mathbf{H_2}$-), 3.3 ppm (2H, m, -N-CH_2CH_2-$C\mathbf{H_2}$-OH), 2.63-2.66 ppm (4H, t, J=6.93, 6.93, 13.87 Hz, -N-$C\mathbf{H_2}$-CH_2-COO-), 2.42-2.35 ppm (6H, m, -OOC-$C\mathbf{H_2}$- and -N-$C\mathbf{H_2}$-), 1.6 ppm (8H, br, -OCH_2-$C\mathbf{H_2}C\mathbf{H_2}$-$CH_2O$-), and 1.51-1.45 ppm(2H, m, -N-$CH_2C\mathbf{H_2}$-CH_2-OH)

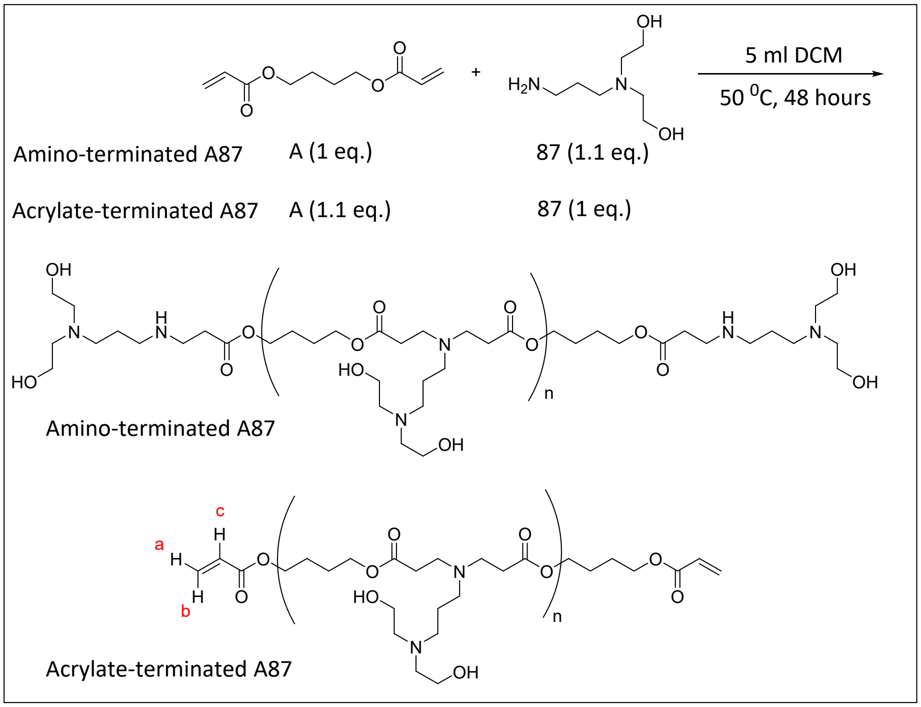

Figure S. 2: The synthesis of amino and acrylate-terminated ***A87***

The ^{1}H-NMR of the acrylate-terminated **A87** 6.3 ppm (2H, d, J= 17.65 Hz, CH_a $\mathbf{H_b}$=CH_c-), 6.1 ppm (2H, dd, J= 10.23, 10.23, 17.65 Hz, CH_a H_b=C$\mathbf{H_c}$-), and 5.9 ppm (2H, d, J= 10.23 Hz, C$\mathbf{H_a}$ H_b=CH_c), 4.0 ppm (8H, br, -COO-C$\mathbf{H_2}$-), 3.44-3.37 ppm (2H, m, -N-CH_2-C$\mathbf{H_2}$-OH), 2.6-2.7 ppm (4H, t, J= 6.76, 6.76, 13.52 Hz, -N-C$\mathbf{H_2}$-CH_2-COO-), 2.43-2.33 ppm (6H, m, -OOC-C$\mathbf{H_2}$- and -N-C$\mathbf{H_2}$), 1.6 ppm (8H, br, -OCH_2-C$\mathbf{H_2}$C$\mathbf{H_2}$-CH_2O-), 1.48-1.41 ppm (2H, m, -N-CH_2C$\mathbf{H_2}$-CH_2-N-).

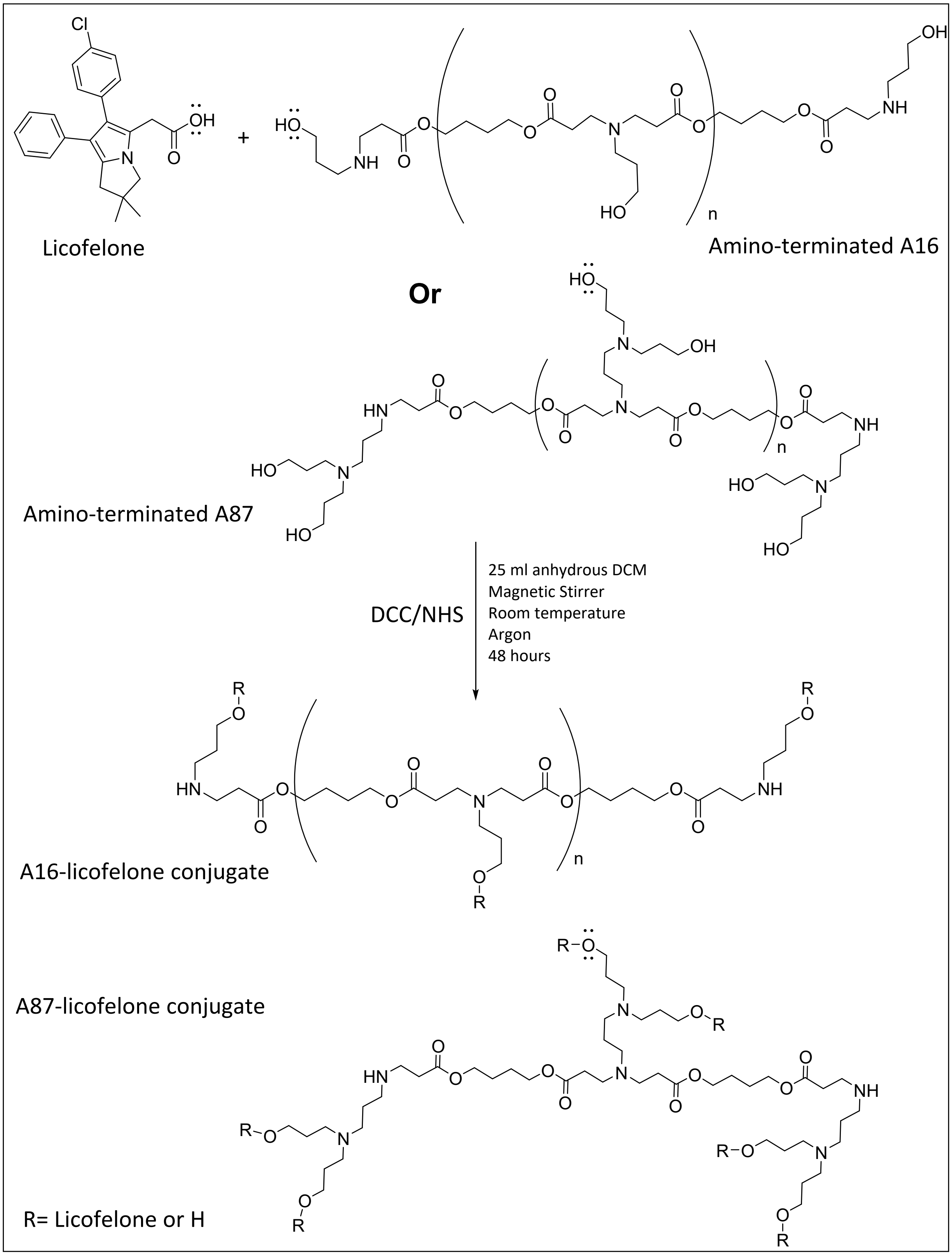

Figure S.3: The reaction mechanism of licofelone with amino-terminated A16

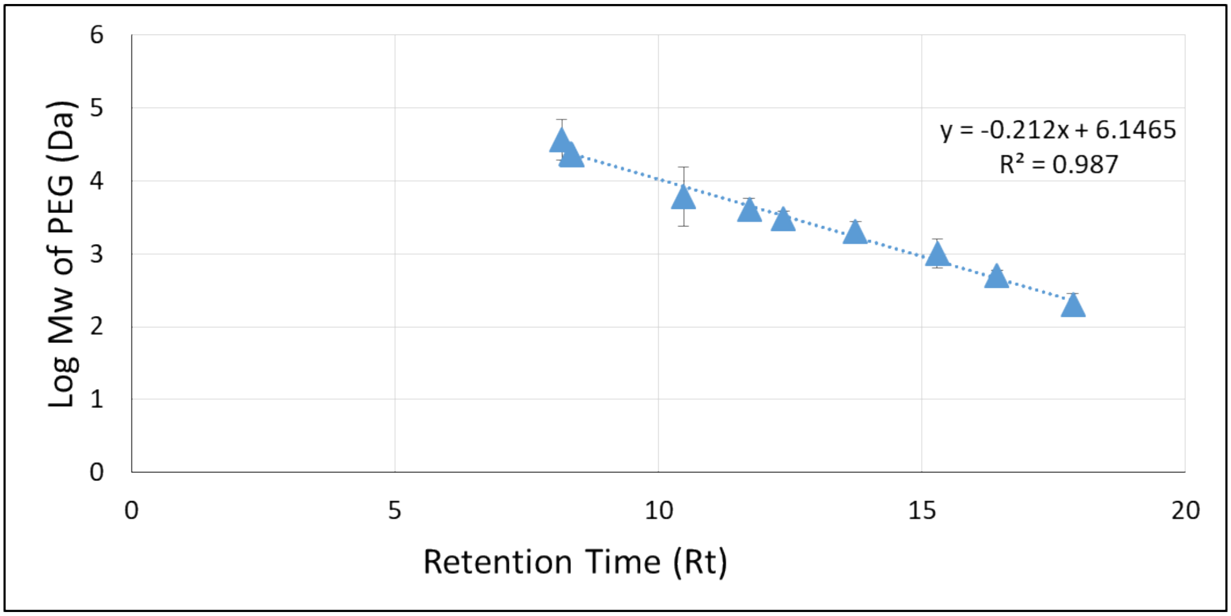

Figure S.4: The calibration curve of polyethylene glycol standards

Bars represent (Mean ± SD of n=2)

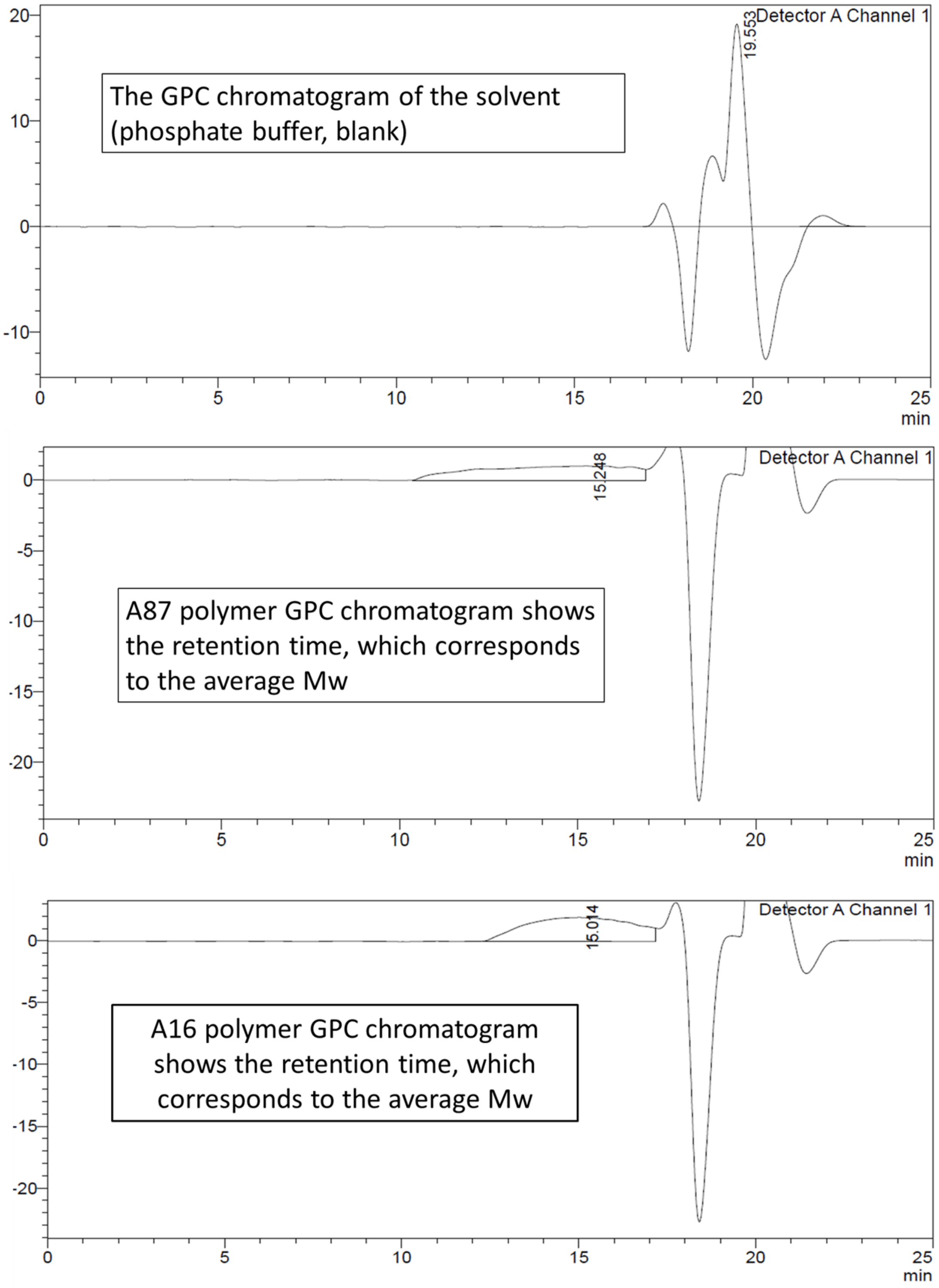

Figure S. 5: The GPC chromatograms of the solvent, A87, and A16 polymers.

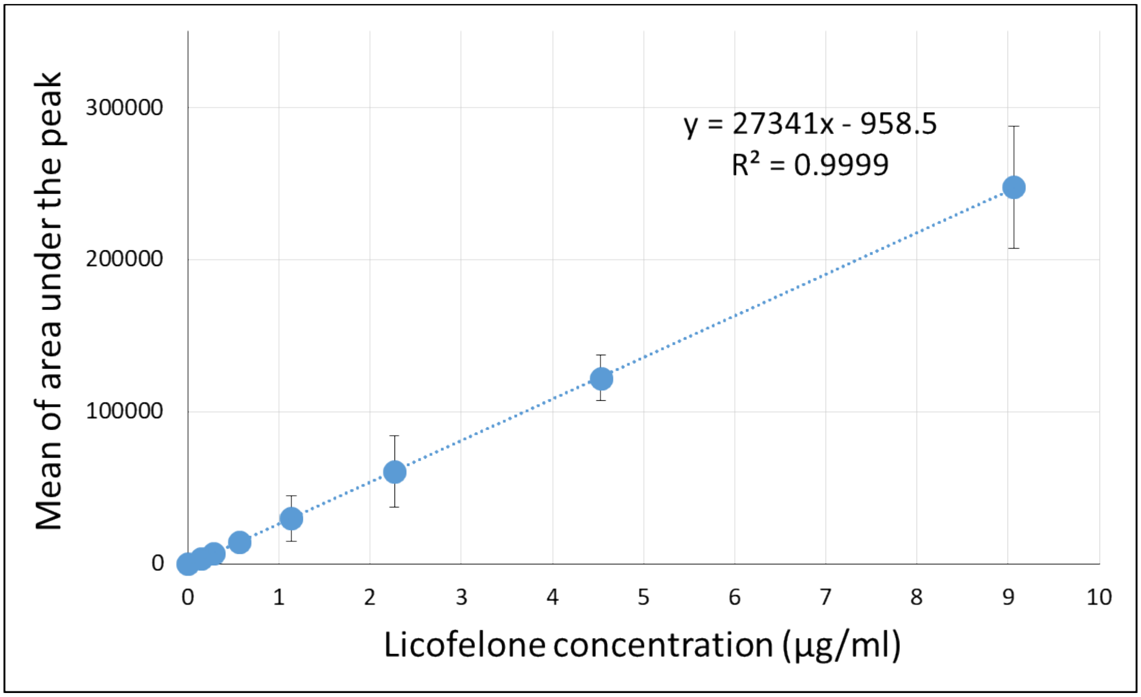

Figure S. 6: The calibration curve of licofelone

Bars represent (Mean ± SD of n=2)

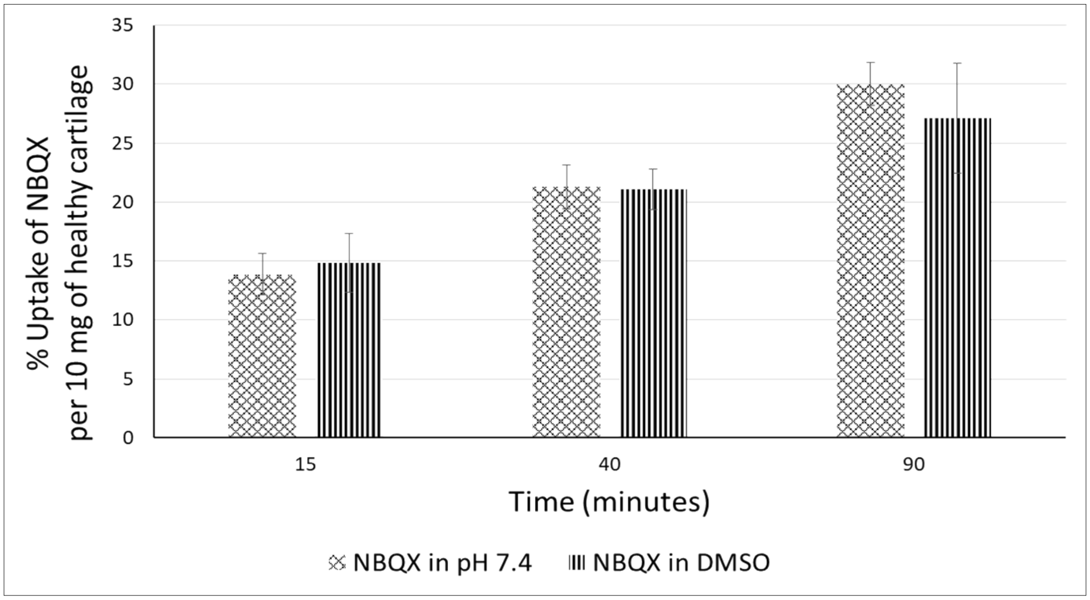

Figure S. 7: The percent uptake of NBQX per 10 mg of healthy cartilage that was dissolved in either PBS pH 7.4 or DMSO. Bars represent (Mean ± SD of n=3).

A healthy cartilage uptake of 0.95 mg/ml of NBQX salt in PBS pH 7.4 or NBQX hydrate in DMSO was observed to determine the effect of DMSO on the drug uptake. The reason for this is that DMSO must be used in licofelone studies since the licofelone is not soluble in aqueous buffer. There was no effect observed between the uptake of NBQX dissolved in PBS and DMSO. Both NBQX were purchased from Sigma Aldrich.

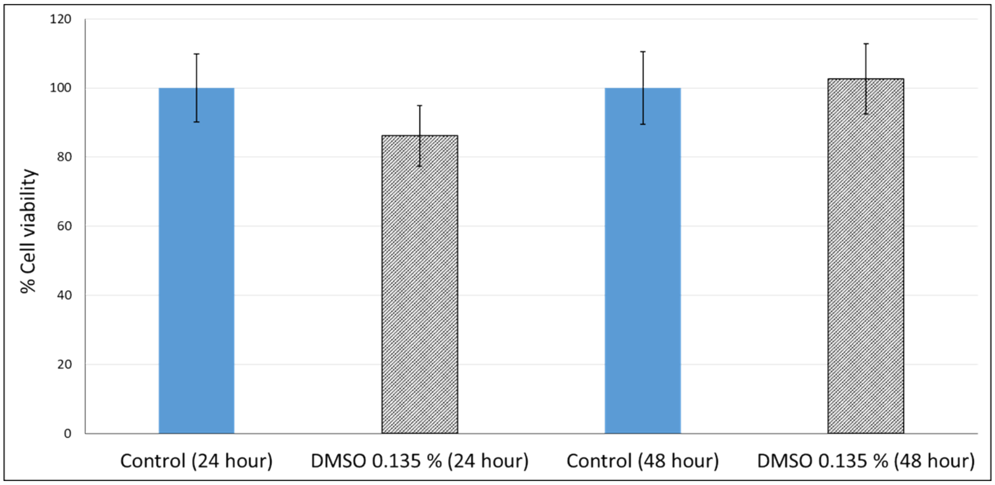

Figure S. 8: The effect of 0.135% DMSO on cell viability. Bars represent (Mean ± SD of n=3)

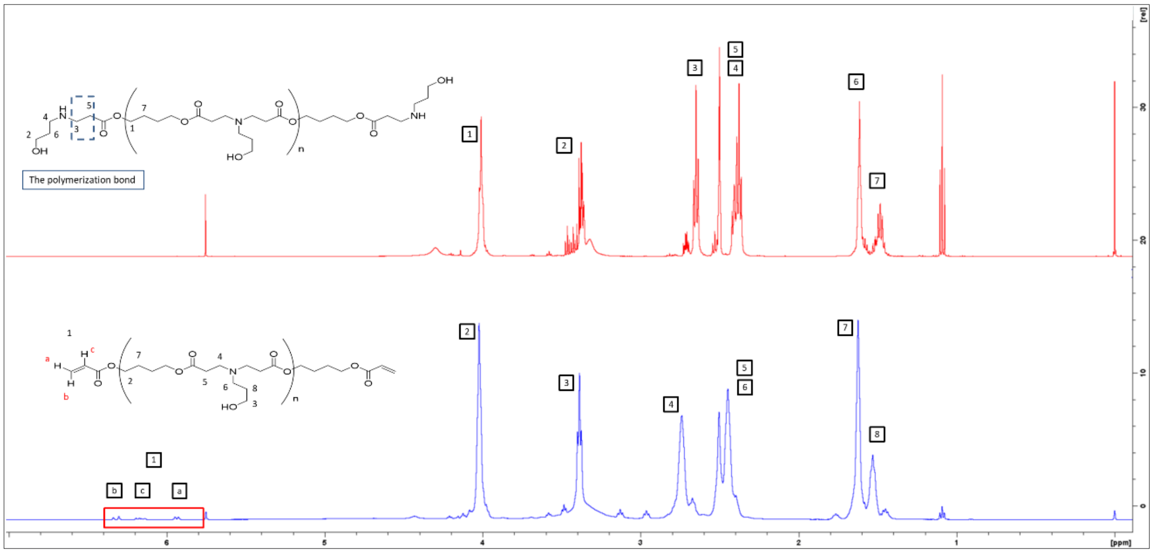

Figure S. 9: The ^{1}H-NMR spectra (500 Hz, DMSO-d_6) of amino and acrylate terminated A16

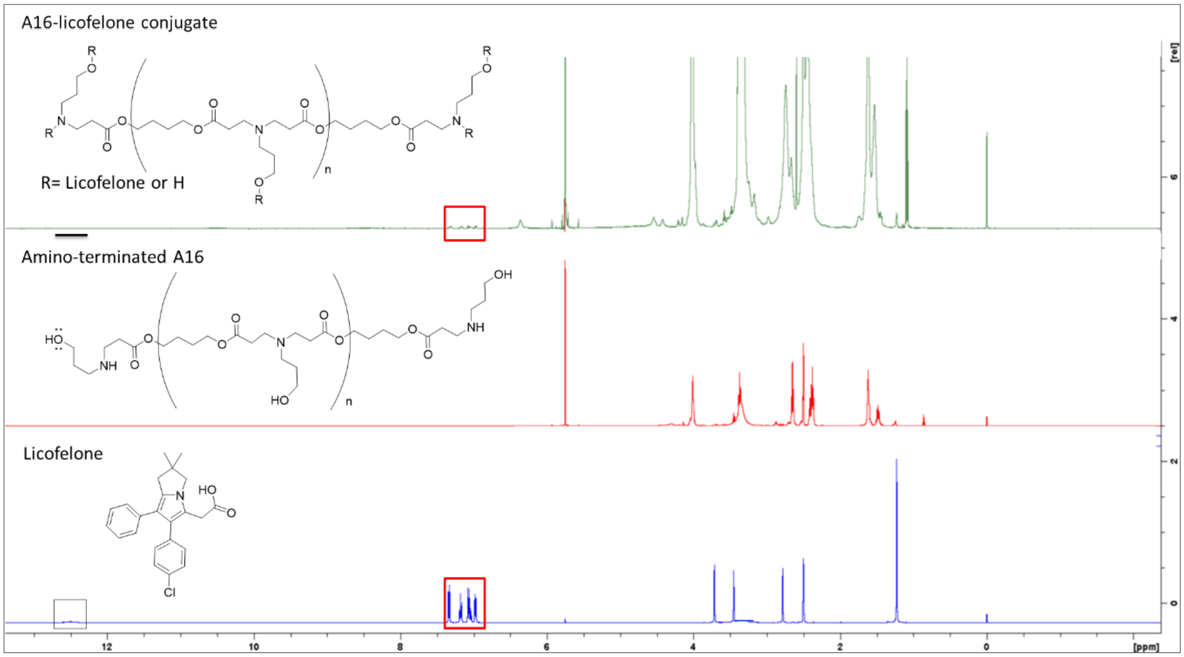

Figure S. 10: The ^{1}H-NMR spectra (500 Hz, DMSO-d_6) of A16-licofelone conjugate, amino terminated A16, and licofelone. The appearance of the licofelone aromatic ring proton peaks (red rectangle) and the disappearance of the carboxylic acid proton (black rectangle) confirm the conjugate formations.

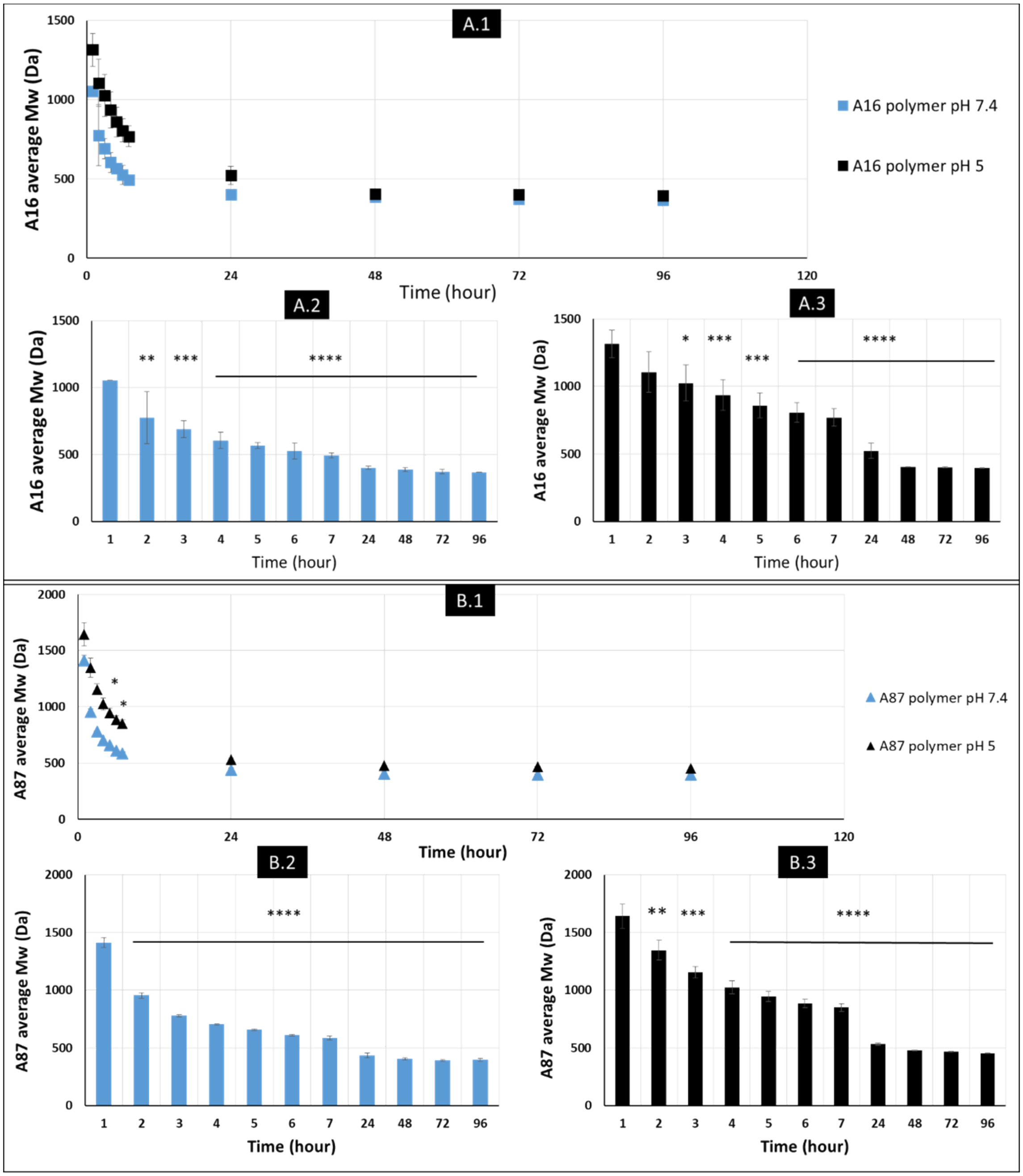

Figure S. 11: The hydrolysis of A16 and A87 at pH 7.4 and pH 5; The A graphs represent the hydrolysis study of A16. The blue (■) and bars represent the hydrolysis at pH 7.4, and the black (■) and bars represent the hydrolysis at pH 5 (mean ± SD of n=3). The B graphs represent the hydrolysis study of A87. The blue (▲) and bars represent the hydrolysis at pH 7.4, and the black (▲) and bars represent the hydrolysis at pH 5 (mean ± SD of n=3). The A.1 and B.1 graphs compare the Mw at pH 7.4 and pH 5 with the respect to the time points of A16 and A87, respectively. The A.2 and B.2 graphs compare the average of Mw at 1 hour to other time points at pH 7.4. The A.3 and B.3 graphs compare the average of Mw at

1 hour to other time points at pH 5. Significant * (P<0.05), ** (P<0.01), * (<0.001), **** (P<0.0001).**

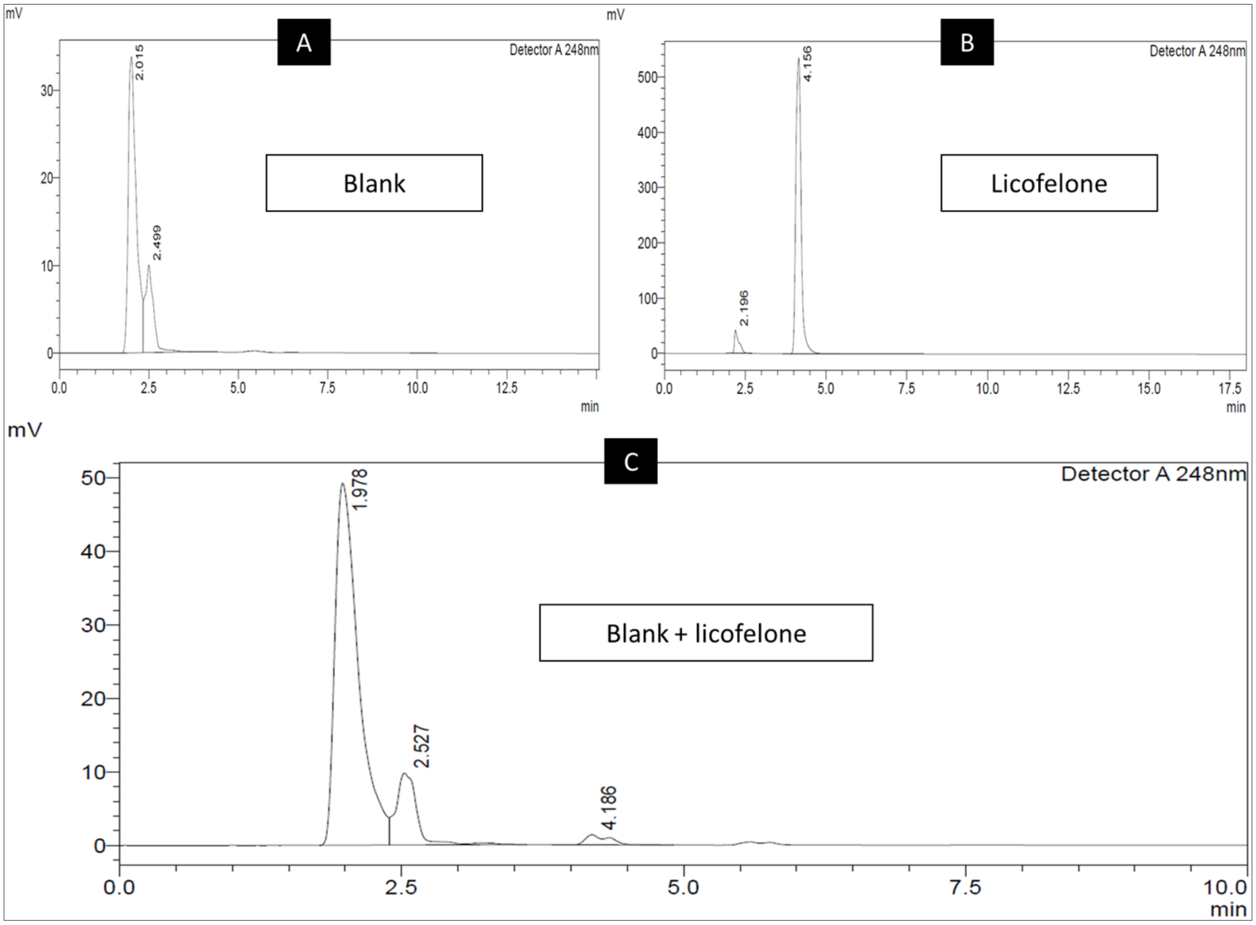

Figure S. 12: The RP-HPLC chromatograms of licofelone

A) RP-HPLC chromatogram shows components involve in the uptake and retention time study, B) RP-HPLC chromatogram of licofelone alone (Rt=4.1 min), and C) RP-HPLC chromatogram shows the separation of licofelone retention time from other components (Rt=4.1 min).

Reference

1. Farndale RW, Buttle DJ, Barrett AJ. Improved quantitation and discrimination of sulphated glycosaminoglycans by use of dimethylmethylene blue. Biochimica et biophysica acta. 1986;883(2):173-7.
2. Saeedi T, Prokopovich P. Poly beta amino ester coated emulsions of NSAIDs for cartilage treatment. Journal of materials chemistry B. 2021;9(29):5837-47. doi: 10.1039/d1tb01024g
